# Editorial: The Human Microbiota in Periodontitis

**DOI:** 10.3389/fcimb.2022.952205

**Published:** 2022-06-27

**Authors:** Marcela Hernández, Marcia Pinto Alves Mayer, Julien Santi-Rocca

**Affiliations:** ^1^ Laboratory of Periodontal Biology and Department of Oral Pathology and Medicine, Faculty of Dentistry, University of Chile, Santiago, Chile; ^2^ Department of Microbiology, Institute of Biomedical Sciences, University of São Paulo, São Paulo, Brazil; ^3^ Science and Healthcare for Oral Welfare, Toulouse, France

**Keywords:** microbiota, periodontitis, gingivitis, oral microbiota, holobiont

Periodontitis is one of the most common diseases in the world, though one of the least understood at the pathophysiological level. The bittersweet interactions between the host and its periodontal microbiota are variable along with time and space and are of central interest for the development of the disease. Keystones pathogens and less known organisms together with inflammation can modify the periodontal ecology and may make this environment acquire traits leading to disease development or exacerbation. These candidates belong to all the kingdoms of the living and their role is yet to be defined, as reviewed by Sedghi et al.

The identification of the pathobionts and patho-modulators of the periodontal biofilm is an essential step in the development of novel prophylactic, diagnostic, and therapeutic strategies targeting periodontitis. By evaluating saliva samples, Diao et al. reported that the degree of dysbiosis increased with disease severity, from periodontal health to gingivitis and periodontitis. These authors suggested that a higher abundance of *Prevotella intermedia* and *Catonella morbi* and a lower abundance of *Porphyromonas pasteri, Prevotella nanceiensis*, and *Haemophilus parainfluenzae* could be biomarkers of periodontitis. In addition, they had shown that a pathogenic microbial community was detected in the saliva of periodontitis patients even after non-surgical periodontal treatment. In an attempt to associate the microbiome with the salivary immune profile, Kawamoto et al. (2021) revealed that the abundance of the potential candidate pathogens and other less-studied organisms was correlated to increased levels of inflammatory mediators whereas the subgingival abundance of *R. aeria* and *H. parainfluenzae* was associated with protective mediators such as CCL-2. Pallos et al. compared the salivary microbiome of subjects with healthy implants to those with peri-implantitis. Dysbiosis was associated with bleeding on probing indicating that inflammation is a major factor in driving shifts of the oral microbiome. No previously recognized pathogen was associated with the salivary microbiome of peri-implantitis patients. However, the genera *Stenotrophomonas*, *Enterococcus*, and *Leuconostoc* were more abundant in participants with peri-implantitis when compared with those with healthy peri-implant sites. Also, the microbiome from the root canal and extraradicular biofilm associated with persistent apical periodontitis was characterized by Sun et al. The dominant phyla were *Proteobacteria, Firmicutes, Bacteroidetes*, and *Actinobacteria*, and the genera *Fusobacterium*, *Morganella, Porphyromonas, Streptococcus*, and *Bifidobacterium;* Nevertheless, in the presence of sinus tracts they contained significantly different microbial communities, which included higher levels of *Porphyromonas, Eubacterium, Treponema*, and *Phocaeicola*, whilst *Microbacterium* and *Enterococcus* were more abundant in the absence of sinus tracts.

It is also intriguing whether the oral microbiome changed over time. Shiba et al. aimed to compare the bacterial composition of the oral microbiome in skeletons of the Japanese Edo era (1603–1867) and modern human samples. Interestingly the microorganisms associated with periodontitis differed between the two populations. *Actinomyces oricola* and *Eggerthella lenta* appeared to have played a key role in periodontitis in the Edo era, whereas known pathogens of the modern era such as *Porphyromonas gingivalis and Fusobacterium nucleatum* subsp. *vincentii* were not.

The association of a certain organism with the disease may not be interpreted as a pathogenic potential. Thus, once identified, the biological characteristics and properties of different microorganisms, such as their virulence factors and modes of action should be studied in different ecological contexts. The interactions among the microorganisms of different kingdoms such as *Candida* and bacteria are determinants of the transition from health to periodontitis, as described by Satala et al. Even the well-studied commensal *Streptococcus* species can alter their transcription profile in an inflammatory environment, possibly contributing to disease progression. By using paired metagenomics and metatranscriptomics, Belstrøm et al. (2021) characterized and compared the transcriptional activity of predominant *Streptococcus* species in different oral sites in health and periodontitis. Their findings revealed that higher transcriptional activity of several *Streptococcus* species is seen under healthy conditions whereas, despite their prevalence, their transcriptional activity decreased under the inflammatory conditions of periodontitis. Other organisms of the Lactobacillales order might be used as probiotics and provide an alternative therapy for periodontitis. Such is the case of the probiotic candidate bacterium *Limosilactobacillus (Lactobacillus) fermentum* ALAL020, which produces a cyclic dipeptide with antibacterial activity against *P. gingivalis* and *Prevotella intermedia*, as reported by Kawai et al.

The role of the microbiome on bone destruction was further discussed by Jia et al. They evaluated the links between gut microbiota and periodontitis, with a particular focus on the underlying mechanisms of the gut-bone axis by which systemic diseases or local infections contribute to bone homeostasis. These authors pointed out evidence showing that not only the oral microbiome but also the gut microbiome could influence bone metabolism and contribute to alveolar bone destruction. Kawamoto et al. (2021) showed that dysbiosis in patients with periodontitis was not restricted to the oral cavity, but was also seen in feces, since the fecal samples of periodontitis were enriched with several Firmicutes, including the families *Lactobacillaceae, Clostridiaceae, Peptostreptococcaceae*, and *Veillonellaceae* whereas the abundance of *Oscillospiraceae* was increased in the fecal samples of healthy subjects.

Several systemic diseases were previously associated with periodontitis, and the identification of microbial signatures in the oral microbiome or other related factors may provide biomarkers, either in serum or in saliva, that distinguish these conditions from health. Among the known periodontal pathogens, *Fusobacterium nucleatum* is involved locally in halitosis and dental pulp infection while it can systemically promote the development or progression of oral cancer and several diseases. Chen et al. discuss the epidemiological evidence, possible pathogenic mechanisms, and therapeutic targeting of *F. nucleatum.*

Regarding the systemic markers, the serological levels of C-reactive protein (CRP), lipopolysaccharide (LPS), and IgG against periodontal pathogens were evaluated in patients with abdominal aortic aneurysm (AAA) by Salhi et al. Antibodies against *P. gingivalis* and *A. actinomycetemcomitans*, LPS, and high CRP concentrations were found in all AAA patients. However, only CRP levels were associated with AAA stability, being higher in unstable AAA, suggesting its predictive value. Though systemic antibiotics have effects on periodontal inflammation and oral microbiota composition, serum and saliva IgG and IgA antibodies against *A. actinomycetemcomitans*,*P. gingivalis*, *P. endodontalis*, *P. intermedia*, and *T. forsythia* remained unchanged as reported by Kopra et al.

Biomarkers in saliva could be associated with different conditions, related to periodontitis or with associated systemic conditions. Saliva is easy to obtain and may reflect the status of the whole oral cavity. The salivary microbiome of participants with obstructive sleep apnea (OSA) was investigated by Chen et al. The microbial community structure differed from the OSA group to the non-OSA group, and OSA was statistically associated with *Peptostreptococcus* and *Granulicatella*.

Most of the studies on the human microbiome have used 16S rRNA sequences analysis. However, this technique has some limitations that were sagaciously shown by RegueiraIglesias et al. This *in silico* study evaluated the coverage of thirty-nine primer pairs currently used in the evaluation of *Bacteria* and *Archaea* communities. They have shown that taxa from different species and even from higher taxonomic levels could be grouped in the same possible OTU, limiting the comparability of the microbial diversity findings reported in oral microbiome literature.

Beside prokaryotes, the eukaryotes *Entamoeba gingivalis* and *Trichomonas tenax* are linked to periodontal diseases, as shown by Yaseen et al. Interestingly, their differential association with gingivitis suggests that this condition should not be considered a low-grade periodontitis. Furthermore, their unequal prevalence in periodontitis reminds us of the existence of different types of periodontal diseases, evidenced here by samples with one, the other, both, or none of these parasite species, but not limited to them.

Overall, the studies on microbiome should not only focus on species identification but rather show the real picture of the pathogenic potential of the microbial community for each patient. To improve microbiome-based preventive and treatment strategies, our knowledge should go beyond microbial signatures evidencing pathogens and integrate the local and systemic interactions among different members of the microbial community, as well as with the host’s immune response in the context of their genetic background. The articles in this special issue “The Human Microbiota in Periodontitis” highlight that unexpected links between periodontitis parameters and other diseases or infections can be drawn if the study design and analysis allow it. It urges us to rethink the pathophysiology models for periodontitis and the concept of holobiont in health and disease.

## Author Contributions

All authors listed have made a substantial, direct, and intellectual contribution to the work and approved it for publication.

## Conflict of Interest

JS-R is employed by Science and Healthcare for Oral Welfare, a private company that raises funds for research in science.

The remaining authors declare that the research was conducted in the absence of any commercial or financial relationships that could be construed as a potential conflict of interest.

## Publisher’s Note

All claims expressed in this article are solely those of the authors and do not necessarily represent those of their affiliated organizations, or those of the publisher, the editors and the reviewers. Any product that may be evaluated in this article, or claim that may be made by its manufacturer, is not guaranteed or endorsed by the publisher.
